# Proteoglycan 4 is Increased in Human Calcified Aortic Valves and Enhances Valvular Interstitial Cell Calcification

**DOI:** 10.3390/cells9030684

**Published:** 2020-03-11

**Authors:** Gonzalo Artiach, Miguel Carracedo, Till Seime, Oscar Plunde, Andres Laguna-Fernandez, Ljubica Matic, Anders Franco-Cereceda, Magnus Bäck

**Affiliations:** 1 Department of Medicine, Karolinska Institutet, 17177 Stockholm, Sweden; gonzalo.artiach@ki.se (G.A.); miguel.carracedo.ortiz@ki.se (M.C.); oscar.persson@ki.se (O.P.); andres.laguna.fernandez@gmail.com (A.L.-F.); 2 Department of Molecular Medicine and Surgery, Karolinska Institutet, 17177 Stockholm, Sweden; till.seime@ki.se (T.S.); ljubica.matic@ki.se (L.M.); Anders.Franco-Cereceda@ki.se (A.F.-C.); 3 Theme Heart and Vessels, Division of Valvular and Coronary Disease, Karolinska University Hospital, 17177 Stockholm, Sweden

**Keywords:** proteoglycan 4, aortic valve stenosis, calcification

## Abstract

Aortic valve stenosis (AVS), a consequence of increased fibrosis and calcification of the aortic valve leaflets, causes progressive narrowing of the aortic valve. Proteoglycans, structural components of the aortic valve, accumulate in regions with fibrosis and moderate calcification. Particularly, proteoglycan 4 (PRG4) has been identified in fibrotic parts of aortic valves. However, the role of PRG4 in the context of AVS and aortic valve calcification has not yet been determined. Here, transcriptomics, histology, and immunohistochemistry were performed in human aortic valves from patients undergoing aortic valve replacement. Human valve interstitial cells (VICs) were used for calcification experiments and RNA expression analysis. PRG4 was significantly upregulated in thickened and calcified regions of aortic valves compared with healthy regions. In addition, mRNA levels of PRG4 positively associated with mRNA for proteins involved in cardiovascular calcification. Treatment of VICs with recombinant human PRG4 enhanced phosphate-induced calcification and increased the mRNA expression of bone morphogenetic protein 2 and the runt-related transcription factor 2. In summary, PRG4 was upregulated in the development of AVS and promoted VIC osteogenic differentiation and calcification. These results suggest that an altered valve leaflet proteoglycan composition may play a role in the progression of AVS.

## 1. Introduction

Inflammation, thickening, and calcification of the aortic valve is known as aortic valve sclerosis. When aortic valve calcification causes left ventricular outflow obstruction, it is referred to as aortic valve stenosis (AVS). AVS is a common disease, with a prevalence of 2–7% in populations above 65 years old [1]. The incidence and severity of AVS increase with age [2] as well as with several cardiovascular risk factors such as obesity [3], smoking [4], and renal dysfunction [5].

The aortic valve is composed of three distinct extracellular matrix (ECM) layers. Closer to the aortic side is the fibrosa, formed by valvular interstitial cells (VICs) and collagen. The middle layer is the spongiosa, composed of VICs and proteoglycans. Finally, closest to the ventricular side is the ventricularis, formed of VICs and elastin [6]. Under homeostasis, VICs promote the maintenance of ECM components, thus providing the structural support and pliability necessary for normal function of the aortic valve. However, inflammation and VIC trans-differentiation promote the abnormal processing of collagen [7] and elastin [8], leading to the characteristic thickening and calcification of the aortic valve causing AVS [9]. Unlike collagen and elastin, less is known about the role of proteoglycans in the pathogenesis of AVS. Proteoglycans are negatively charged molecules capable of promoting lipid retention [10] as well as transforming growth factor beta (TGF-β) retention [11], a cytokine known to promote VIC trans-differentiation [12]. Observational studies have revealed that regions with moderate calcification exhibit an expansion of the proteoglycan content towards the fibrosa layer of the valve [13] and that these proteoglycans tend to accumulate in the surroundings of calcified nodules [14]. Importantly, these observations have been confirmed with proteomics, revealing that fibrotic parts of the valve present higher levels of proteoglycans [15], specifically proteoglycan 4 (PRG4). Furthermore, PRG4 upregulation has been identified in calcified atherosclerotic plaques [16], suggesting a relationship between PRG4 and cardiovascular calcification. Given the link between valvular proteoglycans and calcification, the aims of the current study were to determine the expression of PRG4 in the aortic valve and its role in VIC mediated calcification.

## 2. Materials and Methods

### 2.1. Patients

Human aortic valves were obtained from 88 patients undergoing aortic valve replacement surgery. All patients gave informed consent, and the study was approved by the local ethics committee (2012/1633). N = 64 valves were used for mRNA isolation, N = 4 was used for immunohistochemistry, and N = 20 was used for cell isolation. Immediately after surgical removal, the valves were immersed in either RNA Later (Qiagen, Hilden, Germany) or cell culture media and stored at 4 °C until transport to the laboratory. The study was approved by the local ethics committee (Regionala Etikprövingsnämnden i Stockholm) with approval number 2012/1633 to Professor Anders Franco-Cereceda and was in agreement with the Declaration of Helsinki. All patients gave informed consent.

### 2.2. RNA Extraction and Quality Assessment

Macroscopic dissection was performed dividing each valve into healthy, thickened, or calcified regions as previously described [17]. In brief, healthy tissue was defined as macroscopically transparent and pliable tissue not affected by calcification. Thickened tissue was defined as nontransparent pliable tissue. Calcified tissue was defined as non-pliable, nontransparent, and visually calcified tissue. Different parts were collected from all valve cusps and stored at −80 °C. Total RNA from tissue and cells of human aortic valves was isolated with the RNeasy Lipid Tissue Mini kit (Qiagen, Hilden, Germany), which includes QIAzol Lysis Reagent for lysing tissues to maximize purification. RNA concentration was assessed using a NanoDrop (Thermo Scientific, Waltham, MA, USA), and RNA quality from tissue was evaluated using a 2100 Bioanalyzer (Agilent, Santa Clara, CA, USA). Only high-quality samples with RNA integrity number between 7 and 10 were subsequently employed in the experiments. Cell RNA reverse transcription was performed with High Capacity RNA-to-cDNA Kit (Thermo Fisher Scientific, Waltham, MA, USA). Valve gene expression data from tissue was obtained using Gene Chip Affymetrix human transcriptome 2.0 (HTA 2.0 arrays, Santa Clara, CA, USA) and normalized with Signal space transformation-robust multi-chip analysis (SST-RMA) and log2-transformed using Expression Console (Affymetrix, Santa Clara, CA, USA) as previously described [18]. All recommended quality control assessments were carried, without resulting in exclusion of samples. The following Affymetrix probes were used in the study: TC01001603.hg.1 for PRG4, TC20000067.hg.1 for bone morphogenetic protein 2 (BMP2), TC06000621.hg.1 for runt-related transcription factor 2 (RUNX2), TC04000474.hg.1 for dentin matrix acidic phosphoprotein 1 (DMP1), TC04000475.hg.1 for integrin binding sialoprotein (IBSP), and TC07000137.hg.1 for interleukin 6 (IL6).

### 2.3. Valve interstitial Cells (VICs) Isolation 

Human aortic valves were immersed in cell culture medium (Dulbecco’s Modified Eagle Medium (DMEM), 10% fetal calf serum, 100 units mL^−1^ penicillin, 100 µg mL^−1^ streptomycin, 1 mM sodium pyruvate, 10 mM HEPES, and 2 mM L-glutamine) (Gibco, Waltham, MA, USA) immediately after aortic valve replacement surgery. Next, valves were digested with an enzymatic cocktail composed by collagenase I (2 mg mL^−1^) and dispase II (3.5 mg mL^−1^) (Sigma, St. Louis, MO, USA) for 16 h. The digested valve was then passed through a 0.2-µm filter, and the flow-through was centrifuged at 400 *g* for 5 min. Finally, the cell pellet was washed with phosphate-buffered saline (PBS), centrifuged and resuspended in cell culture medium, and plated in a T150 flask. The phenotype of cells isolated using this protocol is characterized by vimentin and α-smooth muscle actin positivity while being negative for desmin as well as for markers of endothelial and immune cells. VICs were seeded in tissue culture polystyrene containers (Corning, New York, NY, USA), and the medium was changed every second day. Cells were used between passages 2 and 4 for all experiments.

### 2.4. Valve interstitial Cells (VIC) Treatments and Real-Time PCR

For gene expression analysis, VICs were seeded in 12-well plates at 10,000 cells/cm^2^ (Corning, New York, NY, USA). Cells were cultured in the absence or presence of inorganic phosphate (2.6 mM) (Sigma, St. Louis, MO, USA) and harvested after either 9 days or 24 h. VICs treated with either BMP2 (20 ng/mL) or TGF-β (10 ng/mL) (R&D Systems, Minneapolis, MN, USA) for 24 h were used for determination of PRG4 mRNA levels. The effects of PRG4 on BMP2, RUNX2, and SOX9 mRNA levels were determined after 24-h and 9-day incubation of VICs cultured in the absence or presence of hrPRG4 (10 and 100 μg/mL). At the end of the incubation periods, total VIC RNA was isolated as described above. Real-time PCR was performed on a 7900HT Fast RealTime PCR system (Perkin-Elmer Applied Biosystems, Norwalk, CT, USA) as previously described [19] using Taqman Assay-on-Demand (Thermo Fisher Scientific, Waltham, MA, USA) for PRG4 (Hs00981633_m1), BMP2 (Hs00154192_m1), RUNX2 (Hs00231692_m1). and SRY-Box transcription factor 9 (SOX9) (Hs00165814_m1). Relative mRNA expression of target genes was quantified by the 2-ΔCT method using hypoxanthine phosphoribosyltransferase 1 (HPRT) (Hs02800695_m1) as endogenous control.

### 2.5. In Vitro Calcification 

VICs were seeded at 10,000 cells/cm^2^ in 24-well plates (Corning New York, NY, USA). Calcification was induced by culturing VICs for 9 days in cell culture medium (DMEM, 5% fetal calf serum, 100 units/mL penicillin, 100 µg/mL streptomycin, 1 mM sodium pyruvate, 10 mM HEPES, and 2 mM L-glutamine) supplemented with 2.6 mM inorganic phosphate (Sigma, St. Louis, MO, USA).

Full-length recombinant human PRG4 (rhPRG4) was provided by Lubris BioPharma (Weston, MA, USA) [20,21]. Dimethyl sulfoxide (DMSO) (vehicle) or rhPRG4 (10 µg/mL and 100 µg/mL) were added to the calcification medium and changed every other day. Calcification was assessed by IRDye^®^ 800CW BoneTag™ Optical Probe (Li-cor) according to manufacturer’s protocol. In brief, at 24 h before analysis, cells were incubated with the compound IRDye^®^ 800CW BoneTag™ Optical Probe (Li-cor) at a 1:10,000 concentration. After incubation, cells were washed with PBS and fixed with formaldehyde. Fluorescence was assessed and analyzed with an Odyssey CLx (Li-cor) near infrared imager.

### 2.6. Histology

Four of the human aortic valves were used for histological and immunohistochemical analysis. Calcification was assessed by Alizarin Red staining as previously described [22] in order to distinguish calcified from noncalcified regions. In brief, 2% Alizarin Red was diluted in dH_2_O, filtered and pH was adjusted to 4.1–4.3 with either 10% ammonium hydroxide or HCl. For histology, formaldehyde fixed slides were hydrated, incubated for 2 min in 2% Alizarin Red, dehydrated in acetone, followed by acetone-xylene (1/1 *v/v*) solution, and cleared in xylene. Slides were mounted with a synthetic mounting medium (PERTEX, Histolab, Gothenburg, Sweden). Movat pentachrome staining for proteoglycan identification was performed according to manufacturer’s protocol (kit KSC-MPS-1; Nordic Biosite, Stockholm, Sweden), where proteoglycans are stained in blue, muscle is in red, fibrin is in bright red, reticular fibers are in yellow, elastic fibers are in black, and nuclei are in blue/black.

### 2.7. Immunohistochemistry (IHC)

For staining of aortic valves, IHC reagents were obtained from Biocare Medical (Pacheco, CA, USA). Isotype rabbit IgG was used as negative control. In brief, 10-μm sections were deparaffinized in Tissue Clear and rehydrated in ethanol. For antigen retrieval, slides were subjected to high-pressure boiling in DIVADecloaker heat retrieval solution (pH 6.0) or TE buffer (pH 9.0). After blocking with Background Sniper, primary antibodies diluted in Da Vinci Green solution were applied and incubated at room temperature for 1 h. A double-stain probe-polymer detection kit (Mach 2) containing both alkaline phosphatase and horseradish peroxidase was applied, with subsequent detection using Warp Red (for PRG4) and Vina Green (for BMP2 and RUNX2). All slides were counterstained with Hematoxylin, dehydrated, and mounted in Pertex (Histolab, Gothenburg, Sweden). Images were taken using a Nikon OPTIPHOT-2 microscope equipped with digital camera and NIS-Elements software (Nikon Instruments Inc., Melville, NY, USA). The following primary antibody was used in the study: anti-PRG4 (HPA028523; Sigma, St. Louis, MO, USA).

### 2.8. Statistics

Results are expressed as mean ± standard error of the mean (S.E.M.) or as box-and-whiskers plot (Tukey style plot). Statistical significance of differences was assessed with a paired t-test when comparing two groups and with either a 1-way ANOVA repeated measurements followed by a Tukey post hoc test or a 2-way ANOVA repeated measurements followed by a Holm–Sidak post hoc test for multiple comparisons. Spearman correlation coefficient R was calculated for the associations between different genes. Statistical significance was assigned at *p* < 0.05 * (*p* < 0.01 **, *p* < 0.001 ***, *p* < 0.0001 ****). Statistical analyses were performed using GraphPad Prism 8 (GraphPad Software Inc., San Diego, CA, USA).

## 3. Results

### 3.1. Proteoglycan 4 is Upregulated in the Thickened and Calcified Regions of the Aortic Valve

**Figure 1:**
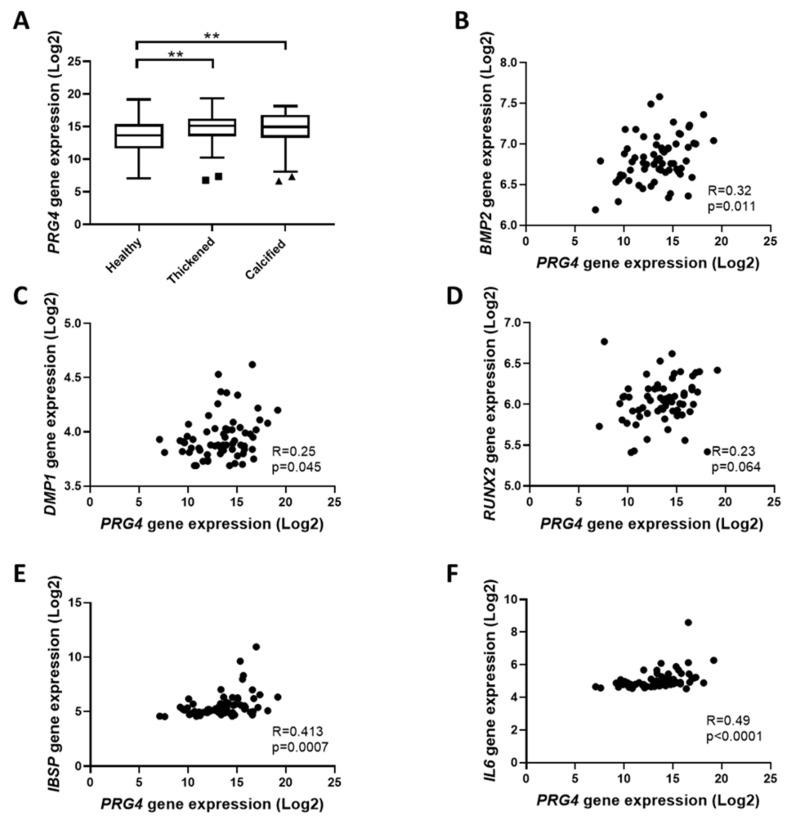
Proteoglycan 4 (PRG4) is overexpressed in the thickened and calcified regions of the aortic valve and associates with calcification related genes: (**A**) Proteoglycan 4 (PRG4) expression from N = 64 patients in healthy, thickened, and calcified regions of the same valve. (**B**–**F**) PRG4 mRNA association in healthy regions (Spearman correlation coefficient; N = 64) with (**B**) bone morphogenetic protein 2 (BMP2), (**C**) dentin matrix acidic phosphoprotein 1 (DMP1), (**D**) runt-related transcription factor 2 (RUNX2), (**E**) integrin binding sialoprotein (IBSP), and (**F**) interleukin 6 (IL6). Statistical significance was determined using a 1-way ANOVA repeated measurements followed by a Tukey post hoc multiple comparisons analysis; ** *p* < 0.01.

PRG4 gene expression in the aortic valve of N = 64 patients undergoing surgical aortic valve replacement was evaluated in healthy, thickened, and calcified tissue within the same valve. Analysis revealed a significantly increased expression of PRG4 in both thickened and calcified tissue of the valve as compared with the healthy tissue in the same valve (Figure 1A), representing 1.9 ± 0.39 and 1.7 ± 0.22-fold increases, respectively. In addition, PRG4 expression in healthy regions of the aortic valve was significantly and positively associated with BMP2, DMP1, IBSP, and IL6, whereas the association with RUNX2 did not reach statistical significance (Figure 1B). The associations with BMP2 and DMP1 were lost in thickened and calcified regions (Table 1).

**Figure 2:**
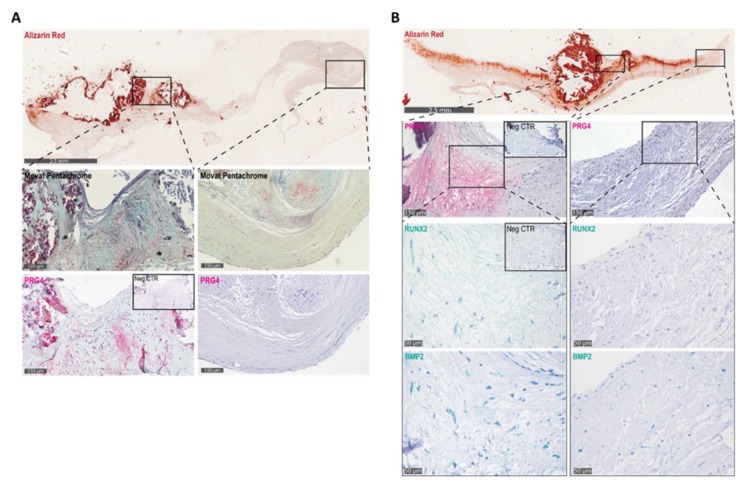
(**A**) Alizarin red staining (red, top panel), proteoglycans by Movat pentachrome (blue, middle panel), and proteoglycan-4 (PRG4) staining (bottom panel) in calcified and noncalcified regions of the aortic valve and (**B**) Alizarin red staining (red, top panel) and immunihistochemical stainings for PRG4, the runt-related transcription factor 2 (RUNX2), and bone morphogenetic protein 2 (BMP2) in calcified and noncalcified regions of the aortic valve.

Interestingly, immunohistochemical analysis in serial human aortic valve sections revealed that PRG4 was expressed in thickened regions with moderate calcification surrounding heavily calcified nodules and in regions corresponding with high proteoglycan deposition as detected by Movat pentachrome staining (Figure 2A). In contrast, areas with low overall proteoglycan deposition were negative for PRG4 (Figure 2A). Furthermore, regions with high PRG4 expression were positive for BMP2 and RUNX2 (Figure 2B).

### 3.2. Proteoglycan 4 Expression in Valvular Interstitial Cells is Upregulated by Pro-Fibrotic and Pro-Calcifying Cytokines

**Figure 3:**
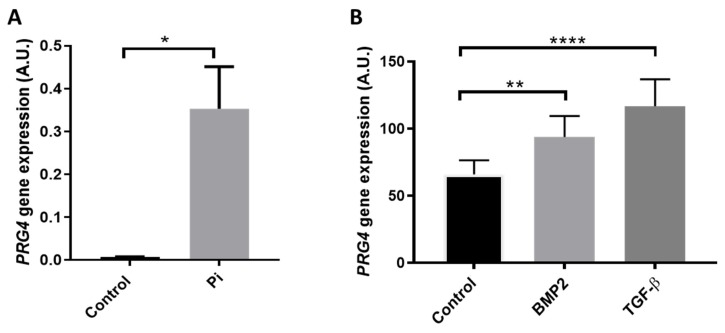
(**A**) Proteoglycan 4 (PRG4) expression in human aortic valvular interstitial cells (VIC) cultured in the absence (control) and presence of 2.6 mM inorganic phosphate (Pi) for 9 days (N = 4) and (**B**) VIC PRG4 expression after 24 h treatment with normal medium (control), bone morphogenic protein 2 (BMP2; 20 ng/mL), and transforming growth factor β (TGF-β ; 10 ng/mL) (N = 9): Statistical significance was determined using a paired t-test (Figure 3A) or a 1-way ANOVA repeated measurements followed by a Tukey post hoc multiple comparisons analysis (Figure 3B); * *p* < 0.05, ** *p* < 0.01, and **** *p* < 0.0001.

To investigate the possible relation between PRG4 expression and calcification of the aortic valve, VICs were treated in vitro with phosphate concentration (2.6 mM) for 9 days. Interestingly, we observed that VIC cultures in the presence of inorganic phosphate exhibited significantly increased PRG4 mRNA levels compared with VICs grown in normal conditions (Figure 3A). To determine which possible factors regulate PRG4 expression in the valve, VICs were treated with BMP2 and TGF-β, observing that both treatments significantly increased PRG4 expression after 24 h (Figure 3B).

### 3.3. Proteoglycan 4 Promotes Expression of Osteogenic Markers and EnhancesCalcificationin Valvular Interstitial Cells.

**Figure 4:**
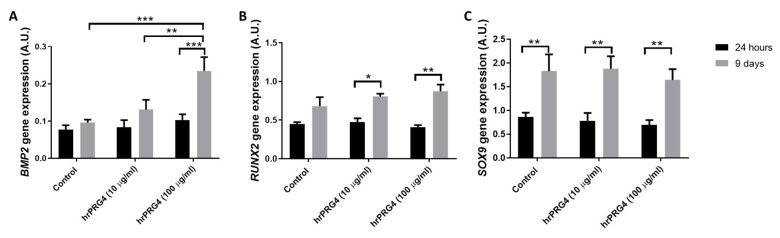
Effects of recombinant human proteoglycan 4 (rhPRG4) on mRNA levels of (**A**) bone morphogenetic protein 2 (BMP2), (**B**) runt-related transcription factor 2 (RUNX2), and (**C**) SRY-Box transcription factor 9 (SOX9) gene expression after 24-h and 9-day culture (N = 3–4): Statistical significance was determined using a 2-way ANOVA repeated measurements followed by a Holm–Sidak post hoc multiple comparisons analysis; * *p* < 0.05, ** *p* < 0.01, and *** *p* < 0.001.

**Figure 5:**
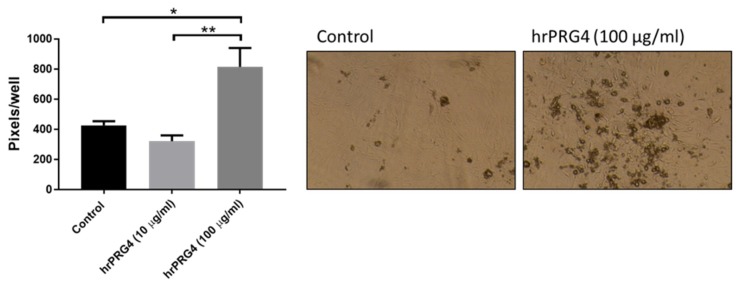
Quantification calcification after 9-day culture of VICs in the presence of either 2.6 mM inorganic phosphate (control) or 2.6 mM inorganic phosphate with addition of recombinant human proteoglycan 4 (rhPRG4) and representative photomicrographs (N = 4): Statistical significance was determined using a 1-way ANOVA repeated measurements followed by a Tukey post hoc multiple comparisons analysis; * *p* < 0.05 and ** *p* < 0.01.

BMP2 mRNA levels were increased by 5.7 ± 1.4-fold after 9-day culture in the presence of compared with the absence of inorganic phosphate (2.6 mM), whereas no significantly changes were observed after 24 h (fold change 1.2 ± 0.11; *p* < 0.05). Treatment of VICs with rhPRG4 (100 µg/mL) for 9 days significantly increased the mRNA levels of BMP2 (Figure 4A). In contrast, the mRNA levels of RUNX2 and SOX9 were not significantly altered by 9-day PRG4 treatment as compared with control (Figure 4B,C). PRG4 did not significantly alter any of the mRNA levels studied at 24 h (Figure 4A–C). Nevertheless, RUNX2 levels were significantly upregulated at 9 days as compared with 24 h only in rhPRG4, whereas RUNX2 mRNA levels were not significantly increased between 24 h and 9 days under control conditions (Figure 4B). In contrast, SOX9 exhibited a time-dependent upregulation both in the absence and presence of rhPRG4 (Figure 4C). In addition, under calcifying conditions, VICs treated with rhPRG4 (100 µg/mL) calcified significantly more compared with control-treated VICs (Figure 5).

## 4. Discussion

In the present study, we show for the first time an increased expression of PRG4 mRNA in the thickened and calcified regions of human aortic valves from 64 patients and its association with genes involved in cardiovascular calcification and inflammation in early stages of disease. PRG4 protein further localized at and in the proximity of valvular calcification processes. In line with our results, PRG4 expression is increased in calcified atherosclerotic plaques, specifically in plaques from symptomatic patients [16]. This increase in PRG4 in calcified valvular and vascular tissues taken together with an upregulation of PRG4 mRNA levels in VICs in response to high phosphate conditions, BMP2, and TGF-β suggests a possible role of PRG4 in VIC differentiation and calcification. Indeed, PRG4 enhanced phosphate induced VIC calcification in the present study. Our results further showed that treatment of VICs with rhPRG4 increased the expression of BMP2 at 9 days of culture. Importantly, the latter was observed also in the absence of high-phosphate supplementation, suggesting that the observed effect of PRG4-induced VIC osteogenic activation was direct rather than a result of increased calcification.

In human aortic valves, PRG4 protein expression localized not only in the proximity of calcifications but also in regions expressing BMP2 and RUNX2 and in areas with high content of overall proteoglycan deposition, a marker of chondrogenesis. Furthermore, treatment with TGF-β, which promotes chondrogenic activation of VICs, [23], significantly increased PRG4 expression. This result is in line with previous studies showing that TGF-β increases PRG4 expression in chondrocytes [24] and promotes glycosaminoglycan elongation of proteoglycans in VICs [25]**.** The latter increased the VIC affinity for low-density lipoprotein which may potentially promote disease progression [25]. However, treatment of VICs with rhPRG4 did not alter the mRNA expression of SOX9, which is a chondrogenic transcription factor [26], and it cannot be excluded that the role of PRG4 in VIC chondrogenesis may be indirect.

It is worth noting that only the higher dose of rhPRG4 used in this study (100 µg/mL) and not the lower dose (10 µg/mL) induced VIC osteogenic activation and calcification, suggesting that PRG4-mediated effects in VICs are tightly regulated and that the osteogenic activation may need a high level of PRG4 expression. Our results are in line with the observed effects of another proteoglycan (biglycan) which promotes RUNX2 expression and VIC calcification through the induction of BMP2 and TGF-β [23].

In summary, PRG4 was upregulated in the development of AVS and promoted VIC osteogenic differentiation and calcification. These results suggest that an altered valve leaflet proteoglycan composition may play a role in the progression of the disease.

## Figures and Tables

**Table 1:** Proteoglycan 4 (PRG4) mRNA association in thickened and calcified regions (Spearman correlation coefficient; N = 64) with bone morphogenetic protein 2 (BMP2), dentin matrix acidic phosphoprotein 1 (DMP1), runt-related transcription factor 2 (RUNX2), integrin binding sialoprotein (IBSP), and interleukin 6 (IL6).

**Thickened Tissue**					
PRG4 vs	BMP2	RUNX2	DMP1	IBSP	IL6
Rho	0.045	0.069	0.209	0.341	0.367
p	0.722	0.589	0.098	0.006	0.003
**Calcified Tissue**					
PRG4 vs	BMP2	RUNX2	DMP1	IBSP	IL6
Rho	0.005	0.112	0.087	0.200	0.125
p	0.971	0.378	0.494	0.113	0.327
